# Listening in Spatialized Noise–Sentences (LiSN-S) as a Measure of Auditory Function in Friedreich Ataxia

**DOI:** 10.1007/s12311-026-02000-7

**Published:** 2026-04-24

**Authors:** Shahd Al Haj Ali, Jessica Early, Jennifer M. Farmer, Sarah Gelbard, Louise Corben, Gary Rance, David R. Lynch

**Affiliations:** 1 Children’s Hospital of Philadelphia, 502 Abramson, CHOP, Philadelphia, PA 19104 USA; 2 Friedreich Ataxia Research Alliance, Downingtown, PA 19335 USA; 3 Bruce Lefroy Centre for Genetic Health Research, Murdoch Children’s Research Institute, Parkville, Melbourne, Australia; 4 Department of Pediatrics, University of Melbourne, Parkville, Australia; 5 Turner Institute for Brain and Mental Health, School of Psychological Sciences, Monash University, Clayton, VIC Australia; 6 Department of Audiology and Speech Pathology, The University of Melbourne, Melbourne, Australia

**Keywords:** Friedreich ataxia, Hearing difficulties, Auditory dysfunction, Binaural

## Abstract

Auditory dysfunction is a clinically significant component of Friedreich ataxia (FRDA), but its features are not readily captured in standard audiology. We examined the Listening in Spatialized Noise–Sentences (LiSN-S) test as a measure of auditory function in a large cohort of subjects with this disease. Methods: We examined 101 individuals with FRDA with the LiSN -S, compared the results with 38 controls, and correlated results with disease features. Results. Control participants scored better than individuals with FRDA on all of the LiSN-S sub-scores. Correlations of LiSN-S scores with the Friedreich Ataxia Rating Scale (FARS) exams were moderate (0.5–0.7), while correlations were lower with genetic markers such as GAA1 and age of onset (0.32–0.54). Correlations were lower still with disease duration (0.25–0.29). Linear regressions to define the independent effects of age and GAA1 demonstrated that GAA1 uniformly predicted LiSN-S advantage scores (p < 0.01) but age predicted spatial advantage and total advantage ( P < 0.01) but not talker advantage. LiSN-S scores were slightly more affected in individuals with clinical auditory complaints. Conclusions: LiSN -S testing captures audiologic dysfunction in FRDA in a manner that appears to dependent on genetic severity and to a lesser degree time.

## Introduction

Friedreich’s Ataxia (FRDA) is a rare autosomal recessive neurodegenerative disorder caused by homozygous GAA trinucleotide repeat expansions in the *FXN* gene, which encodes the mitochondrial protein frataxin [1, 2]. Although typical clinical manifestations include progressive gait ataxia, sensory neuropathy, and hypertrophic cardiomyopathy, auditory dysfunction represents an important yet under-recognized aspect of the disease [3–5]. Many individuals with FRDA report difficulty hearing and understanding speech in noisy environments despite normal sound detection levels, suggesting the presence of central auditory processing abnormalities that can impact communication, learning, and overall quality of life [3].

The auditory dysfunction in FRDA reflects changes in both peripheral and central pathways. The spiral ganglion neurons degenerate early based on pathological and electrophysiological studies, paralleling the loss of the dorsal root ganglia, olfactory neurons, and retinal ganglion cells in FRDA [6–8]. Brainstem auditory evoked potentials (BAEP) are abnormal [9] with individuals with less severe FRDA typically showing reduced response amplitudes and those with more advanced disease presenting with absent waveforms [10]. Still, the functional consequences of auditory dysfunction in FRDA remain insufficiently understood.

Most prior investigations have focused on aural neural conduction or physiological measures with limited attention to how such abnormalities translate into real-world listening difficulties [11, 12]. The Listening in Spatialized Noise–Sentences (LiSN‑S) test provides an assessment of speech-in-noise perception by evaluating the listener’s ability to use spatial and voice cues to segregate competing speech streams. Unlike conventional audiometric testing, LiSN-S advantage scores isolate central auditory processing mechanisms while minimizing the influence of cognitive factors [13]. In the present study, we characterize speech-in-noise perception in a large cohort of individuals with FRDA using the LiSN-S and compare their performance with healthy controls. Furthermore, we explore associations between auditory performance and markers of disease severity, including the Friedreich Ataxia Rating Scale (FARS) scores and GAA1 (GAA repeat size on smaller allele) repeat length [14, 15]. We hypothesized that participants with FRDA would demonstrate impaired use of spatial and talker cues, consistent with auditory neuropathy–related deficits in temporal and binaural processing, and that the magnitude of these impairments would correlate with both clinical and genetic severity.

## Patients and Methods

### Participants

Participants were recruited from the Friedreich Ataxia Clinical Outcome Measure Study (FACOMS) natural history study (NCT03090789, registered 2017-03-27). All procedures performed in studies involving human participants were in accordance with the ethical standards of the institutional and/or national research committee and with the 1964 Helsinki Declaration and its later amendments or comparable ethical standards. The protocol was approved by the Institutional Review Board at the Children’s Hospital of Philadelphia (#2609). All participants, or their legal guardians, provided written informed consent. This work was funded by a grant to DRL from the Friedreich Ataxia Research Alliance.

All individuals with FRDA had biallelic FXN mutations (pathogenic GAA repeat expansions or the presence of a pathogenic point mutation/deletion). Healthy control participants were recruited from relatives of individuals with FRDA and other sources. As part of FACOMS, detailed clinical and genetic data were already available for all participants with FRDA, including GAA repeat lengths for both alleles (GAA1 and GAA2), age at symptom onset, disease duration, presence of auditory symptomatology (a single dichotomous question in FACOMS), and the FARS scores.

### Functional Hearing Assessment

All participants completed the LiSN-S test in a quiet room with participants wearing calibrated headphones that delivered spatialized, three-dimensional signals. The LiSN-S software presents target sentences alongside competing speech maskers that vary in voice (same or different speaker) and spatial location (0° or ± 90° azimuth). For each of the four listening conditions—Same Voice at 90° (SV90), Different Voice at 90° (DV90), Same Voice at 0° (SV0), and Different Voice at 0° (DV0)—the speech reception threshold (SRT) was determined. SRT is the signal-to-noise ratio (SNR) required for 50% correct sentence identification, expressed in decibels (dB). From these four baseline conditions, the software calculates three advantage scores by subtracting SRTs across specific condition pairs: Talker Advantage, Spatial Advantage, and Total Advantage.

### Statistical Analysis

Descriptive statistics were calculated for demographic, clinical, and auditory variables using R version 4.5.1, or in STATA version 18. For participants with repeated LiSN-S assessments, only the most recent data were used for cross-sectional analyses. Between-group comparisons (FRDA vs. controls) were conducted using Mann–Whitney U tests due to non-normal distributions, with effect sizes calculated using rank-biserial correlation. Values are shown as mean *±* standard deviation for variables with normal distributions and medians with IQR for non-normal variables. Associations between auditory outcomes (LiSN-S scores) and disease-related variables (FARS scores, smaller GAA repeat lengths (GAA1), disease duration, and age at symptom onset) were explored using Spearman’s rank correlations. We also compared such values after converting the LiSN results to Z-scores, to account for the relative lack of control subjects at pediatric ages.

Z scores were created as described previously accounting for age using the formulas and normative data from Cameron et al. [16] placed in a Microsoft Exce calculator apopliication:

1. *Low and High Cue SRT*: mean = max (intercept + b * age, max[c, d + e * age]).

Cutoff score= mean + (2 * SD of the residuals from the age-corrected trend lines).

2. *Talker, Spatial, and Total Advantage*: mean = min (intercept + b * age, c).

Cutoff score = mean – (2 * SD of the residuals from the age-corrected trend lines).

DV0 and SV90 Z score were calculated separately using the formulas:

1 $$\mathrm D \mathrm V0_z = (-9.83+19.6*\mathrm e\mathrm x\mathrm p(-age/6.74) - \mathrm D \mathrm V0)/2.4$$

2 $${\mathrm S \mathrm V90_z} = (-16.03 + 27.5*\mathrm e\mathrm x\mathrm p(-Age/4.16) -- SV90)/1.79$$

Significant or near-significant associations identified in the correlation matrix (particularly FARS and GAA1) were further examined using linear regression models (to allow simultaneous consideration of variables of age and GAA1, which are linked in based on disease severity). Separate models were constructed for each LiSN-S outcome, with one model depending on FARS and the other on GAA1 and age as predictors. A significance threshold of *p* < 0.05 was used throughout.

Stratified analysis: We also compared LiSN outcome values in analyses stratified above or below the mean GAA1 (A), above and below age 18 (B), or both (C). Mean values were calculated are compared by t test between the strata for each of these stratifications. For stratification by age, values were also compared to control values.

## Results

### Participant Characteristics

One hundred and one individuals with FRDA and 38 healthy control participants were included in the analysis. Controls were significantly older ( *P* < 0.0068) than individuals with FRDA, but within control subjects there was no correlation of LiSN-S scores with age (highest correlation *r* = 0.14 for talker ad, *P* = 0.10). Controls and individuals with FRDA were not significantly different by sex. Mean disease duration among participants with FRDA was 14.4 ± 9.9 years, with a mean FARS score of 67.0 ± 18.8 and mean GAA1 repeat length of 674 ± 212 (Table 1), consistent with a diverse cohort of participants at mid to early disease stage.

**Table 1:** Participant demographics and clinical characteristics (mean ± SD)

Variable	Friedreich’s ataxia (101)	Control (38)
Age (years)	25.9 ± 13.3 (range 7–68)	33.5 ± 17.4 (range 11–68)
Female, n (%)	63 (62.4%)	21 (55.3%)
Disease Onset (years)	11.6 ± 6.8 (range 3–39)	-
Disease Duration (years)	14.6 ± 10.6 (range 1–42)	-
GAA1 Repeat Length	674 ± 212 (range 136–1000)	-
GAA2 Repeat Length	927 ± 220 (range 280–1400)	-
FARS Score	67 ± 18.8 (range 31-97.5)	-

We also assessed which participants had complaints of auditory symptomatology. 51% of participants with FRDA had such complaints; such individuals were of similar age (26.0 *±* 15.7 vs. 26.9 *±* 17.7) but had slightly longer mean GAA1 values (707 *±* 209 vs. 650 *±* 213; *p* = 0.19), though this was not significant.

### LiSN-S Performance

**Fig. 1:**
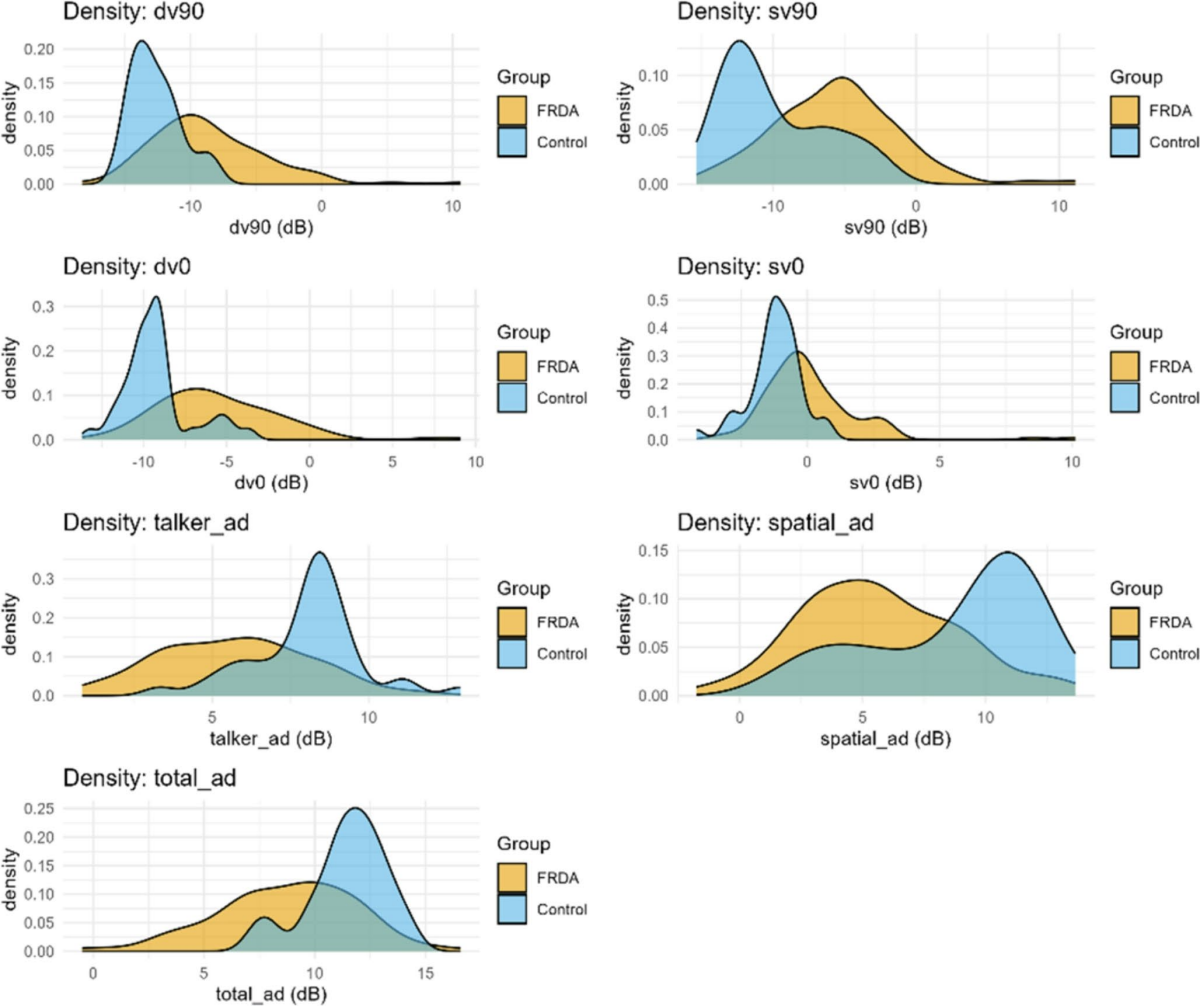
Histogram of LiSN-S scores in control individuals and participants with FRDA. FRDA values differed from control values, though the 2 distributions overlapped

**Fig. 2:**
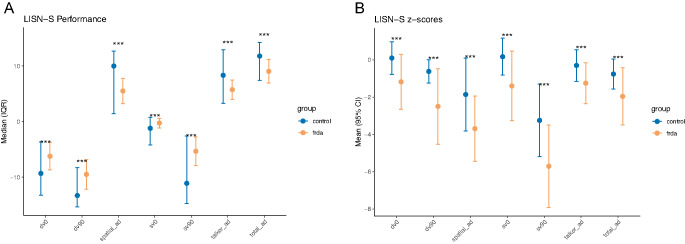
LiSN-S performance: group comparisons. Values were compared across LiSN-S tests for control (blue) and FRDA subjects ( yellow). Mean + SD shpownb for each point. A=raw scores, B= Z scores normalized for age [17]. ***Mann-Whitney U test SV90=same voice at 90° , DV90=different voice at 90°, SV0=same voice at 0°,DV0= different voice at 0° . Talker ad =talker advantage, Spatial ad =spatial advantage, Total ad =total advantage

Compared with controls, individuals with FRDA demonstrated reduced performance (higher speech-reception thresholds and reduced spatial/talker advantage) across all LiSN-S derived scores, though there was overlap between the groups and the effects sizes were between (0.35 and 0.55) consistent with such overlap (Fig. 1). Median scores for Talker Advantage, Spatial Advantage, and Total Advantage were all lower in the FRDA group using both raw scores (Fig. 2A) and Z scores (Fig. 2B). Multiple LiSN-S outcomes demonstrated moderate correlations with FARS, lower correlations with the genetic markers GAA1 and age of onset, and still lower magnitude correlations with disease duration (Table 2). Correlations were positive for primary LiSN measures but negative for advantage measures. In linear regression models including both age and GAA1 as independent variables, GAA1 significantly predicted all LiSN-S variables while age failed to predict DV0 and Talker advantage (Table 3). Correlations of age with LiSN-S values in control subject were all less than 0.15 except for DV0 (0.22, *p* = 0.22).

**Table 2:** Spearman correlation coefficient between LiSN-S values and disease features

FARS	DV90	DV0	SV90	SV0	Talker ad	Spatial ad	Total ad
	0.65 *p* < 0.001	0.60 *p* < 0.001	0.67 *p* < 0.001	0.54 *p* < 0.001	-0.51 *p* < 0.001	0.58 *p* < 0.001	-0.59 *p* < 0.001
GAA1	0.47 *p* < 0.001	0.47 *p* < 0.001	0.40 *p* < 0.001	0.34 *p* < 0.001	-0.44 *p* < 0.001	-0.34 *p* < 0.001	-0.44 *p* < 0.001
Onset	-0.45 *p* < 0.001	-0.54 *p* < 0.001	-0.38 *p* < 0.001	-0.32 *p* = 0.002	0.52 *p* < 0.001	0.32 *p* < 0.001	0.40 *p* < 0.001
Duration	0.29 *p* = 0.004	0.16 *p* = 0.12	0.27 *p* = 0.008	0.25 *p* = 0.013	-0.17 *p* = 0.098	-0.22 *p* = 0.035	-0.29 *p* = 0.005

*SV90*  Same Voice at 90°, *DV90*  Different Voice at 90°, *SV0 *Same Voice at 0°, *DV0* Different Voice at 0°. *Talker ad* Talker Advantage, *Spatial ad* Spatial Advantage, *Total ad* Total Advantage. GAA1 = length of shorter GAA length, *FARS* Friedreich Ataxia Rating Scale. Spearman’s – spearman correlation coefficient

**Table 3:** LiSN-S individual outcome linear regression models using age/GAA1 or FARS

	DV90		SV90		DV0		SV0		**Talker advantage**		**Spatial advan**tage		**Total advantage**	
	Coeff *+ SE*	*p*-value	Coeff *±* SE	*p*-value	Coeff *±* SE	*p*-value	Coeff*±* SE	*p*-value	Coeff+	*p*-value	Coeff	*p*-value	Coeff+SE	*p*-value
GAA1	0.012 *±* 0.002	< 0.001	0.010 *±* 0.002	< 0.001	0.009 *±* 0.002	0.000	0.001	< 0.001	-0.006+0.001	<0.001	-0.005+0.001	0.00	-0.008+0.001	<0.001
Age	0.091 *±* 0.030	0.003	0.080 *±* 0.030	0.009	0.033 *±* 0.026	0.217	0.033 *±* 0.013	0.012	0.001+ 0.018	0.991	-0.049+0.023	0.03	-0.058+0.022	0.008
FARS	0.16 *±* 0.02	0.001	0.152 *±* 0.020	< 0.001	0.131 *±* 0.018	0.00	0.061 *±* 0.009	< 0.001	-0.070+0.013	<0.001	-0.095+0.015	0.00	-0.100+0.014	<0.001

LiSN-S individual outcome linear regression models were created using either Age and GAA1 or FARS scores. *SV90 *Same Voice at 90°, *DV90 *Different Voice at 90°, *SV0 *Same Voice at 0°, *DV0 *Different Voice at 0°. GAA1= length of shorter GAA length, *FARS *Friedreich Ataxia Rating Scale

Finally, we further defined the relationship between age, GAA1 length and LiSN-S scores using stratified analysis (Table 4A, B, C). LiSN -S scores (reflecting higher speech-reception thresholds and reduced spatial/talker advantage) were significantly more abnormal in individuals with GAA1 lengths above the cohort mean (674) than those below the cohort mean. Those with GAA1 lengths shorter than the mean were still significantly different than control values on all LiSN-S parameters. In contrast, there were small differences in scores for individuals above and below age 18, of which only talker advantage reached significance. In addition, the scores among individuals with GAA1 lengths above 674 did not differ significantly between those above and below age 18, suggesting that major determinant of LiSN-S scores is GAA1 length rather than progression across age.

**Table 4:** A, B, C. Stratification of LiSN-S results by GAA1 (A), age (B) or (C) age and GAA1

A.				
Parameter	Control (mean *±* SD) (*n* = 38)	FA GAA1 < 675 (Mean *±* SD) (*n* = 42)	FA GAA1 *≥* 675 (Mean FA *±* SD) (*n* = 58)	
DV90	-12.7 *±* 1.9	-10.6 *±* 3.3 (*p* = 0.0009 vs. control)	-7.21 *±* 4.88 (*p* < 0.0001 vs. GAA1 < 675)	
SV90	-9.9 *±*3.6	-7.03 *±* 3.58 (*p* = 0.0006 vs. control)	-3.93 *±*4.76 (*p* = 0.0006 vs. GAA1 < 675)	
SV0	-1.21 *±* 0.97	-0.60 *±* 1.21 (*p* = 0.015 vs. control)	0.81 *±* 2.13 (*p* = 0.0002 vs. GAA1 < 675)	
DV0	-9.26 *±* 2.03	-7.33*±* 2.43 (*p* = 0.0003 vs. control)	-4.20*±* 4.05 (*p* < 0.0001 vs. GAA1 < 675)	
talker ad	8.06 *±* 1.79	6.73*±* 1.93 (*p* = 0.0021 vs. control)	5.01 *±* 2.36 (*p* = 0.0002 vs. GAA1 < 675)	
spatial ad	8.67 *±* 3.34	6.45 *±* 3.24 (*p* = 0.0035 vs. control)	4.90 *±* 3.08 (*p* = 0.017 vs. GAA1 < 675)	
total ad	11.5 *±*1.75	9.98 *±* 2.85 (*p* = 0.0058)	8.02*±* 3.18 (*p* = 0.002 vs. GAA1 < 675)	
B.				
Parameter	FA age < 18 (Mean *±* SD) (*n* = 31)	FA age *≥* 18 (Mean *±* sd) (*n* = 70)	Control, age < 18 (Mean *±* SD) (*n* = 6)	Control age *≥* 18 (Mean *±* sd) (*n* = 32)
Dv90	-8.46 *±* 3.18	-8.77 *±* 5.09 (*p* = 0.99 vs. < 18 )	-10.9 *±* 2.3	-12.9 *±* 1.65 (*p* = 0.47 vs. < 18)
Sv90	-4.81 + 3.58	-5.50 *±* 4.90 (*p* = 0.99 vs. < 18)	-8.13 *±* 3.81	-10.2 + 3.5 (*p* = 0.20 vs. < 18)
Sv0	0.31 *±* 1.23	0.17 *±* 2.18 (= 0.74 vs. < 18)	-0.88 *±* 0.74	-1.27 *±* 1.01 (*p* = 0.38 vs. < 18)
Dv0	-4.65 *±* 2.76	-6.01 *±* 4.01 (*p* = 0.09 vs. < 18)	-8.49 *±* 3.49	-9.40 *±* 1.67 (*p* = 0.35 vs. < 18)
Talker ad	4.96 *±* 2.01	6.18 *±* 2.43 (*p* = 0.016 vs. < 18)	7.60 *±* 3.38	8.14 *±* 1.39 (*p* = 0.51 vs. < 18)
Spatial ad	5.40 *±* 2.74	5.63 *±* 3.42 (*p* = 0.74 vs. < 18)	7.26 *±* 3.54	8.97 *±* 3.29 (*p* = 0.26 vs. < 18)
Total ad	8.74 *±* 2.96	8.92 *±* 3.37 (*p* = 0.80 vs. < 18)	10.1 +2.25	11.7 *±* 1.54 (*p* = 0.04 vs. < 18)
C.				
Parameter	> 675, age > 18 (32)	> 675 age < 18 (25)		
DV 90	-6.69 *±* 5.84	-7.79 *±* 3.66 (*p* = 0.39 vs. > 18)		
SV 90	-3.77 *±* 5.51	-4.32 *±* 2.48 (*p* = 0.62 vs. > 18)		
SV 0	1.09 *±* 2.57	0.39 *±* 1.22 (*p* = 0.18 vs. > 18)		
DV 0	-4.35 *±* 4.90	-4.11 *±* 2.49 (*p* = 0.81 vs. > 18)		
Talker ad	5.45 *±* 2.77	4.50 *±* 1.64 (*p* = 0.11 vs. > 18)		
Spatial ad	4.87 *±* 3.56	5.09 *±* 2.37 (*p* = 0.78 vs. > 18)		
Total ad	7.79 *±* 3.65	8.38*±* 2.34 (*p* = 0.46 vs. > 18)		

Stratification of LiSN-S results by GAA1 (A) , age (B) or (C) age and GAA1. Results were compared by t test ( above or below GAA1 for A; above or below 18 for B,C). *SV90 *Same Voice at 90°, *DV90* Different Voice at 90°, *SV0 *Same Voice at 0°, *DV0* Different Voice at 0°. *Talker ad  *Talker Advantage, *Spatial ad *Spatial Advantage, *Total ad*  Total Advantage

### Context of LiSN-S Scores and Clinical Symptomatology

**Fig. 3:**
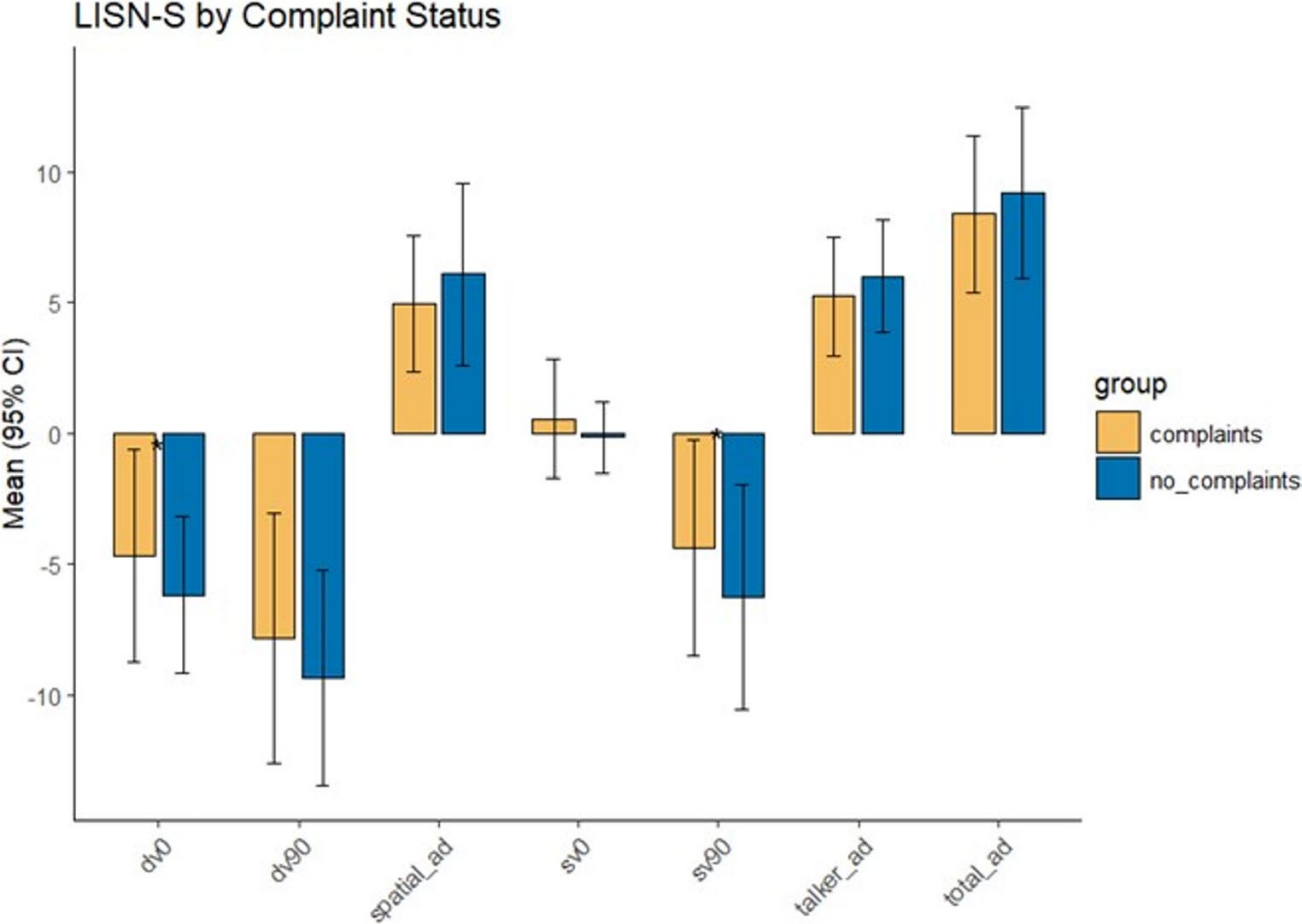
Stratification of LiSN-S scores by hearing symptomatology. Stratification of LiSN-S results by presence or absence of auditory symptomatology. Comparisons were made between those subjects with hearing complaints and those without. SV90 = same voice at 90°, DV90 = different voice at 90°, SV0 = same voice at 0°,DV0 = different voice at 0°. Talker ad =talker advantage, Spatial ad =spatial advantage, Total ad =total advantage. Only SV90 (*p* = 0.029) and DV0 (*p* = 0.039) were significantly different

Finally, we stratified LiSN-S scores based on whether individuals reported auditory dysfunction (Fig. 3). LiSN-S scores differed more from control values numerically in those who reported auditory dysfunction, but such reached only borderline significance compared with scores in those without complaints, suggesting that the auditory dysfunction in FRDA is incompletely captured by LiSN-S testing.

## Discussion

The present study demonstrates a significant difference in LiSN-S test performance between individuals with FRDA and healthy controls along with significant correlations between clinical characteristics and auditory measures. Although the controls differed slightly in age, no effect of age on LiSN-S scores was noted in the control group, and the results were consistent across all methods of analysis, suggesting that such a mismatch cannot explain differences between control and FRDA participants. In all four conditions tested, participants showed impaired perceptual ability consistent with a diminished capacity to track rapid signal changes requiring precise neural timing for accurate understanding. Importantly, the two conditions with spatially separated target speech and noise (DV90 and SV90) were the most affected as they require biaural inputs for accuracy (Table 2). The LiSN-S test was originally designed to evaluate auditory processing in complex, noisy environments. Deviations from normal performance in this test suggest auditory processing difficulties that originate at various levels of the auditory pathway, ranging from cochlear structures to the auditory cortex. In FRDA, auditory dysfunction is not necessarily associated with peripheral hearing impairment despite abnormal auditory brainstem responses (ABR) [5, 11]. As a result of the brainstem involvement, binaural hearing responses (as tested in LiSN-S) are abnormal in FRDA consistent with the abnormal ABR testing.

LiSN-S results are associated with markers of clinical and genetic severity suggesting that auditory pathway involvement occurs in the context of the overall neurodegeneration in FRDA [17]. The effect of genetic markers of degeneration in FRDA on LiSN-S scores was greater than the effect of time (age, disease duration), and the effect of GAA1 repeat length above the cohort average on LiSN-S values was striking compared to values from subjects below the average GAA1. A smaller study noted similar associations with genetic severity and LiSN -S scores [18]. As LiSN-S scores clearly progress based on correlations with age, duration, and functional severity, the effect of time on LiSN -S scores is probably confounded in cross sectional data by the strong effect of GAA1 repeat length. Such strong dependence on genetic severity is also seen in the other severe features of FRDA (cardiomyopathy, diabetes, vision loss, scoliosis) [19–21] even though such a dependence on genetic severity was not as apparent in the presence or absence of auditory symptomatology. The present results also correspond to the evolving concept of FRDA as a disorder with strong presymptomatic genetically-determined developmental components that precede and facilitate later neurodegenerative components [19–23].

The present study substantially reproduces the results of smaller earlier studies, though several differences appear. The difference in LiSN-S scores between controls and participants with FRDA was smaller in this study with overlap between the groups [9]. This likely reflects the apparently higher GAA1 repeat length, lower age of onset, but shorter disease duration of subjects of the previous study. Clinical report of hearing difficulties also was associated with worse LiSN-S scores, consistent with LiSN-S scores marking clinical auditory dysfunction, though this did not reach full significance. Overall, the relative absence of effects of disease duration/age and clinical auditory dysfunction may reflect variability in testing paradigms (either related or unrelated to FRDA). In previous studies, LiSN-S scores have not shown significant practice effects [16]. Thus, longitudinal data may be useful in defining the role of this test in clinical situations.

## Summary

LiSN-S captures auditory dysfunction in FRDA with a particular emphasis on genetic severity.

## Data Availability

Data will be shared upon reasonable request.

## Declarations

### Clinical Trial Registration

N/A ( not a clinical trial) though the FACOMS study is registered as NCT03090789, 2017-03-27,.
